# Pain in Parkinson's disease is impacted by motor complications, anxiety and sleep disturbances

**DOI:** 10.1002/ejp.4765

**Published:** 2024-12-29

**Authors:** Katarina Rukavina, Juliet Staunton, Pavlos Zinzalias, Magdalena Krbot Skoric, Karolina Poplawska‐Domaszewicz, Antonio Pisani, Kirsty Bannister, K Ray Chaudhuri

**Affiliations:** ^1^ Institute of Psychiatry, Psychology & Neuroscience at King's College London London UK; ^2^ Parkinson's Foundation Centre of Excellence King's College Hospital NHS Foundation Trust London UK; ^3^ Department of Neurology University Hospital Center Zagreb Zagreb Croatia; ^4^ Department of Neurology Poznan University of Medical Sciences Poznan Poland; ^5^ Centro Interdipartimentale di Ricerca in Ipertensione Arteriosa e Patologie Associate Federico II University of Naples Naples Italy; ^6^ Federico II University Hospital Naples Italy

## Abstract

**Background:**

Parkinson's disease (PD) is the second most common neurodegenerative disease. Over two thirds of People with Parkinson's (PwP) live with chronic PD‐related pain, but its successful management remains an unmet need. Unrevealing links between pain and other motor and non‐motor symptoms (NMS) of PD may accelerate delivery of much needed precision pain medicine approaches for PwP.

**Methods:**

An exploratory, cross‐sectional analysis of the prospective, observational, multicentre, international study ‘*The Non‐motor International Longitudinal, Real‐Life Study in PD ‐ NILS*’.

**Results:**

In 109 PwP (41.3% women, age 64.29 ± 9.80 years, disease duration 5.50 (2.44–10.89) years, H&Y stage 2 (1–4), levodopa equivalent daily dose 575.00 (315.00–1004.00) mg), strong correlations were noted between the total burden of PD‐related pain and the total NMS burden (*r*_s_ = 0.641) and moderate with disturbances of sleep/fatigue (*r*_s_ = 0.483), cognitive issues (*r*_s_ = 0.445), motor complications (*r*_s_ = 0.421), anxiety (*r*_s_ = 0.441) and depression (*r*_s_ = 0.451). In a multivariate linear regression analysis, motor complications (*B* = 2.063, 95% CI for B 1.152–2.974, *p* < 0.001), sleep disturbances/fatigue (*B* = 0.392, 95% CI for *B* 0.064–0.720, *p* = 0.020) and anxiety (*B* = 0.912, 95% CI for *B* 0.165–1.659, *p* = 0.017) significantly impacted the overall burden of pain.

**Conclusions:**

In PwP, PD‐related pain is significantly impacted by motor complications, anxiety and sleep disturbances. A personalized, tailored approach to management of pain in PwP need to accurately identify and tackle all its interrelated symptoms. Whether successful management of motor complications, anxiety and sleep disturbances may contribute to pain relief in PwP for specified cohorts needs to be a focus of future randomized controlled clinical trials.

**Significance statement:**

This explorative analysis identifies the frequent overlap of chronic pain, motor complications, sleep disturbances and anxiety in Parkinson's disease and could help advance the development of precise and effective pain management strategies tailored to the needs of People with Parkinson's.

## 1 INTRODUCTION

Parkinson's disease (PD) is the second most common neurodegenerative disease, affecting over 8.5 million individuals worldwide; by 2040, its prevalence is expected to increase to 12–17 million people (Collaborators, 2017; epidemiology) (Dorsey et al., 2018). Chronic pain affects over two thirds of People with Parkinson's disease (PwP), diminishes their health‐related quality of life (HRQoL) and considerably restricts their everyday activities (Buhmann et al., 2017; Ford, 2010; Martinez‐Martin et al., 2017). However, its successful management remains hampered by the elusive driving force and multifaceted clinical presentation (Rukavina et al., 2022).

In general, effective pain management requires a thorough understanding of how pain interacts with other comorbidities and how addressing these comorbidities affects pain burden (Pickering et al., 2023). PD is a clinicopathological entity presenting as a unique and evolving set of clinical (motor and non‐motor symptoms, NMS) and biological features in each affected individual (Titova et al., 2017) (Postuma et al., 2015) (Marras et al., 2024). Successful management of PD‐related pain necessitates holistic, patient‐based approach, informed by the interrelations between PD‐related pain and motor and NMS of PD (Pain).

Specific PD‐subtypes have been proposed in an attempt to allow for prediction of disease progression, prediction of treatment responses and precision medicine delivery (Marras et al., 2024). Applying a hypothesis‐driven subtyping, Sauerbier et al. proposed a pain‐dominant PD non‐motor subtype, ‘Park Pain’, arising, in part, from the effects of PD‐specific neurodegeneration on the limbic system, marked by variety of painful syndromes present throughout the course of the disease, often in a manner disproportionate to the severity of motor symptoms (Sauerbier et al., 2016). Later, underpinned by a novel neurotransmitter‐based subtyping concept, Chaudhuri et al. defined the noradrenergic phenotype, whose key feature is PD‐pain, co‐occurring with a set of related NMS, including sleep dysfunction, mood disorders and dysautonomia (Ray Chaudhuri et al., 2023). Using a data‐driven clinical subtyping approach in a cross‐sectional study, Ghosh et al. reported co‐occurrence of pain alongside cardiovascular disturbances and sleep dysfunction, strengthening this hypothesis (Ghosh et al., 2020). Furthermore, evidence from neuroimaging studies indicates the involvement of dopaminergic neurotransmitter signalling pathways in nociceptive processing in PwP and PD‐related pain has recently been linked to the severity of dopaminergic depletion in the caudate nucleus (Rukavina et al., 2023) (Brefel‐Courbon et al., 2005).

Here, we investigated PD‐related pain and its links with motor and NMS of PD using validated clinical assessment tools in a large real‐word dataset, ‘*The Non‐motor International Longitudinal, Real‐Life Study in PD ‐ NILS*’. We hypothesized co‐occurrence of PD‐related pain in a constellation of symptoms suggestive of underlying alterations in noradrenergic and dopaminergic neurotransmitter signalling pathways.

## 2 METHODS

We ran an exploratory, cross‐sectional (one‐point‐in‐time) analysis on the study sample of participants recruited to the prospective, observational, multicentre, international study NILS (authorized by the South‐East London Research Ethics Service, reference number: 10/H0808/141 and adopted by the National Institute of Health Research in the United Kingdom [UK National Institute for Health Research Clinical Research Network, UKCRN, No.: 10084]) at the Parkinson's Foundation Centre of Excellence at King's College Hospital NHS Foundation Trust in London, UK, in the period between 1 July 2020 and 31 December 2022. The NILS study, set up in 2011, includes 34 centres worldwide (across Europe, Asia, United States, Latin America and Australia). Here, clinical assessments with validated tools are performed during routine clinical follow‐up appointments with an aim to evaluate NMS burden and progression in unselected population of individuals with PD, presenting to a clinician in a real‐life setting (van Wamelen et al., 2021). Written informed consent was obtained from all participants. Individuals with a diagnosis of PD (the Brain Bank Criteria) within 5 years since the disease onset were enrolled if no exclusion criteria (atypical parkinsonism, concomitant severe disease, conditions interfering with PD assessments and inability to give informed consent) were present (Hughes et al., 1992) (Rukavina et al., 2023). Individuals with pain caused by any non‐PD‐related condition (as established by their treating neurologists, based on the medical history and clinical examination) were not enrolled (Rukavina et al., 2023).

### 2.1 Assessments

Demographic (age and gender) and disease‐related features (disease duration—DD, current medication) were collected in a structured interview. The Levodopa Equivalent Daily Dose (LEDD) was calculated (Schade et al., 2020). Analgesics were classified into three categories: non‐steroidal anti‐inflammatory drugs (NSAIDs), opioid analgesics and anticonvulsants.

The following clinical assessments from the NILS database were included:

#### 2.1.1 Rater‐based assessments

*King's Parkinson's Disease Pain Scale* (*KPPS*) consists of 14 items, organized into seven domains, corresponding to seven subtypes of pain frequently present in PwP (musculoskeletal [MSK] pain, chronic pain, fluctuation‐related pain, nocturnal pain, orofacial pain, discoloration/oedema/swelling and radicular pain) and inquiries about the pain experienced in the previous month. Each item includes ratings of the severity (on a scale 0–3) and frequency (on a scale 0–4); multiplying the severity and the frequency, a score is calculated for each item, ranging from 0 to 12. Total score is a sum of all item scores (possibly ranging from 0 to 168) (Chaudhuri et al., 2015).

*Hoehn and Yahr (H&Y) stage*—this scale grades patients' degree of disability and general functional level into one of the following five categories: unilateral disease (H&Y Stage I), bilateral disease with intact balance (H&Y Stage II), the presence of postural instability (H&Y Stage III), loss of physical independence (H&Y Stage IV) and being wheelchair‐ or bed‐bound (H&Y Stage V). This practical staging method allows for reproducible assessments by independent examiners (Hoehn & Yahr, 1967).

*Short Parkinson's Evaluation Scale (SPES)/SCales for Outcomes in Parkinson's disease—Motor Function (SCOPA‐Motor)*—is a rater‐completed scale consisting of 21 items, divided into three sections: Motor Evaluation (Section A, 7 items), Activities of Daily Living (Section B, 10 items) and Motor Complications (Section C, 4 items, including dyskinesia and ‘off’ periods). Four response options (0—normal to 3—severe) are offered for each item. A total score is a sum of all items (Martinez‐Martin et al., 2005).

*Non‐motor Symptoms Scale (NMSS)* is a healthcare professional completed scale consisting of 30 items, grouped into nine domains: cardiovascular and falls, sleep/fatigue, mood/apathy, perceptual problems, attention/memory, gastrointestinal, urinary, sexual and miscellaneous (the latter consisting of pain, changes in sense of smell, weight change and hyperhidrosis). Each item score is calculated by multiplying severity (score 0–3) and frequency (score 1–4) over the time frame of the past month. The NMSS total score is a sum of all item scores and may range from 0 to 360 (Chaudhuri et al., 2007).

#### 2.1.2 Patient‐completed outcomes

*Hospital Anxiety and Depression Scale (HADS)* consists of anxiety subscale (HADS‐A) and depression subscale (HADS‐D), each with seven items for self‐rating on a 5‐point scale (0–4) (Zigmond & Snaith, 1983) In each subscale, scores of 5–9 indicate mild anxiety/depression, 10–14 indicate moderate anxiety/depression, 15–19 indicate moderately severe anxiety/depression, and ≥ 20 indicate severe anxiety/depression.

*The Parkinson's Disease Sleep Scale (PDSS)* is a simple patient‐completed questionnaire addressing 15 commonly reported symptoms associated with sleep disturbance in PD, designed as a visual analogue scale—PD patients are asked to place a cross mark on the 10 cm line to mark the severity of their symptoms. To quantify the answer, the rater measures the distance along each line to the intersection with the cross in centimetres (Chaudhuri et al., 2002).

*The Parkinson's Disease Questionnaire—Short Form (PDQ‐8)* is a PD‐specific measure of self‐perceived health status consisting of eight items with five response categories based on how often, due to their PD, PwP have experienced a problem defined by each item, with answers ranging from 0 (‘never’) to 4 (‘always’ or ‘cannot do at all’). All item scores are summed together to calculate the total score (Jenkinson & Fitzpatrick, 2007).

### 2.2 Statistical analysis

Statistical analysis was performed using SPSS Statistics software, version 26.0 (IBM SPSS for Mac, Armonk, NY, USA, IBM Corp.). The normality of the data distribution was estimated (the one‐sample Kolmogorov–Smirnov test), and descriptive statistics provided. The between‐the‐groups comparisons were carried out (the independent samples *t*‐test, Mann–Whitney test or Person's chi‐squared test) and the associations between burden of PD‐related pain and other disease‐related features probed (Spearman's correlation), with Bonferroni correction applied where appropriate. Factors significantly impacting PD‐pain burden in univariate regression models were subsequently entered into a multivariate regression model.

## 3 RESULTS

This analysis presents data from 109 PwP (41.3% women, mean age 64.29 ± 9.80 years, median DD 5.50 [2.44–10.89] years, median H&Y stage 2 [1–4], SCOPA‐Motor scale, total score 17.71 ± 7.69, median LEDD 575.00 [315.00–1004.00] mg) with a median KPPS total score of 15.00 (5.50–27.50) and mean PDQ‐8 total score 9.15 ± 6.09. Participants' demographic and clinical data are displayed in the Table 1.

**TABLE 1:** Patients' demographic and clinical characteristics.

Age (years; mean ± SD)	64.29 ± 9.80
Sex (Female: *n*, %)	45 (41.3%)
Ethnicity (*n*, %)	White 98 (89.9%) Black 7 (6.4%) Asian 0 (0%) Mixed/Other 4 (3.7%)
Disease duration (Years; Median, IQR/Mean ± SD)	5.50 (2.44–10.89)/ 7.32 ± 6.30
Hoehn & Yahr stage (Median, Range, IQR)	2 (1–4; 2–3)
SCOPA‐Motor scale, total score (Mean ± SD)	17.71 ± 7.69
LEDD (mg; Median, IQR/Mean ± SD)	575.00 (315.00–1004.00) 543.08 ± 308.94
KPPS total score (Median, IQR/Mean ± SD)	15.00 (5.50–27.50) 18.60 ± 16.33
PDQ‐8 total score (Mean ± SD)	9.15 ± 6.09
NMSS total (Mean ± SD)	56.56 ± 39.66
NMSS D1 *Cardiovascular, falls* (Median, IQR/Mean ± SD)	0 (0–2) 1.39 ± 2.26
NMSS D2 *Sleep, fatigue* (Median, IQR/Mean ± SD)	10 (3–16.5) 11.5 ± 9.66
NMSS D3 *Mood, cognition* (Median, IQR/Mean ± SD)	4 (0–12) 8.71 ± 11.7
NMSS D4 *Hallucinations* (Median, IQR/Mean ± SD)	0 (0–0) 0.93 ± 2.93
NMSS D5 *Attention, memory* (Median, IQR/Mean ± SD)	2 (0–7.50) 5.83 ± 9.02
NMSS D6 *Gastrointestinal* (Median, IQR/Mean ± SD)	3 (0–7) 4.49 ± 5.1
NMSS D7 *Urinary* (Median, IQR/Mean ± SD)	6 (0.5–12) 8.78 ± 9.7
NMSS D8 *Sexual* (Median, IQR/Mean ± SD)	0 (0–4) 3.25 ± 5.86
NMSS D9 *Miscellaneous* (Median, IQR/Mean ± SD)	12 (5–18) 11.7 ± 8.36
HADS—*Anxiety* (Median, IQR/Mean ± SD)	6 (4–10) 7.06 ± 4.25
HADS—*Depression* (Median, IQR/Mean ± SD)	5 (3–8) 5.95 ± 3.98
PDSS total	98.97 ± 26.68

*Note*: Depending on the normality of distribution, variables are summarized as mean ± standard deviation, or median and interquartile range.

Abbreviations: HADS‐A, Hospital Anxiety and Depression Scale, sub‐score for anxiety; HADS‐D, Hospital Anxiety and Depression Scale, sub‐score for depression; IQR, Interquartile Range; KPPS, King's Parkinson's Disease Pain Scale; LEDD, Levodopa Equivalent Daily Dose; NMSS, Non‐motor Symptoms Scale; PDQ‐8, Parkinson's Disease Questionnaire 8; PDSS, Parkinson's Disease Sleep Scale; SCOPA‐Motor, SCales for Outcomes in Parkinson's disease—Motor Function; SD, standard deviation.

Of all participants, 92.7% declared pain: 79.8% declared MSK pain, 44% fluctuation‐related, 37.6% nocturnal, 34.9% radicular, 24.8% chronic PD‐related pain, 16.5% pain related to discolouration, oedema or swelling, while 6.4% had orofacial pain, as assessed using the KPPS.

Amongst participants with pain (*n* = 101), 51.5% (*n* = 52) were taking pain medication: 47.5% (*n* = 48) utilized NSAIDs, 5.9% (*n* = 6) opioids and 3.0% (*n* = 3) gabapentinoids. Eight participants who did not declare any pain were also taking analgesics: 62.5% (*n* = 5) opioids and 37.5% (*n* = 3) NSAIDs. The highest prevalence of analgesics use (82%) was noted amongst participants with radicular pain.

Significant correlations (Bonferroni correction, *p* < 0.0023) were noted between the total burden of pain (KPPS total score) and other features of PD:

- Strong with total NMS burden (NMSS total score, *r*_s_ = 0.641, *p* < 0.001);
- Moderate with disturbances of sleep/fatigue (NMSS D2: *r*_s_ = 0.483, *p* < 0.001), attention/memory issues (NMSS D5 *r*_s_ = 0.445, *p* < 0.001), motor complications (SCOPA‐Motor Part C: *r*_s_ = 0.421, *p* < 0.001), anxiety (HADS‐A: *r*_s_ = 0.441, *p* < 0.001) and depression (HADS‐D: *r*_s_ = 0.451, *p* < 0.001); and
- Weak with DD (*r*_s_ = 0.382, *p* < 0.001), H&Y stage (*r*_s_ = 0.363, *p* < 0.001), LEDD (*r*_s_ = 0.340, *p* < 0.001), overall burden of motor symptoms (SCOPA‐Motor total score, *r*_s_ = 0.289, *p* = 0.002), mood and cognitive issues (NMSS D3, *r*_s_ = 0.329, *p* < 0.001) and gastrointestinal dysfunction (NMSS D6 *r*_s_ = 0.358, *p* < 0.001).

Higher overall burden of pain correlated significantly with worse HRQoL (PDQ‐8 total) (*r*_s_ = 0.544, *p* < 0.001).

Features strongly or moderately correlating with the overall pain burden were further investigated in a linear regression analysis: In a univariate linear regression analysis, they all demonstrated significant associations with the KPPS total score (Table 2). However, when entered jointly in a multivariate linear regression model, only motor complications, sleep dysfunction/fatigue and anxiety retained statistical significance as determinants of the overall burden of pain (Table 2).

**TABLE 2:** Univariate and multivariate logistic regression models exploring potential determinants of the overall pain burden.

	Univariate linear regression			Multivariate linear regression		
	*B*	95% CI for B	*p*	*B*	95% CI for *B*	*p*
**Overall pain burden (KPPS total score)**						
**SCOPA‐Motor** **Part C**	2.818	1.918–3.718	**<0.001**	2.063	1.152 to 2.974	**<0.001**
**NMSS total**	0.243	0.180–0.307	**<0.001**		n.a.	
**NMSS D2** *Sleep/Fatigue*	0.816	0.532–1.100	**<0.001**	0.392	0.064 to 0.720	**0.020**
**NMSS D5** *Attention/Memory*	0.604	0.277–0.931	**<0.001**	0.083	−0.221 to 0.387	0.590
**HADS‐A**	1.585	0.913–2.256	**<0.001**	0.912	0.165 to 1.659	**0.017**
**HADS‐D**	1.850	1.148–2.552	**<0.001**	0.080	−0.867 to 1.027	0.868

*Note*: Statistically significant associations (Bonferroni correction, *p* < 0.0023) are shown in bold.

Abbreviations: HADS, Hospital Anxiety and Depression Scale; KPPS, King's Parkinson's Disease Pain Scale; NMSS, Non‐motor Symptoms Scale; SCOPA‐Motor, SCales for Outcomes in Parkinson's disease—Motor Function.

Next, PD‐related pain was clinically subtyped (using the KPPS), and participants were separated into groups based on the presence or absence of each subtype of the PD‐related pain. The severity of motor complications, sleep dysfunction/fatigue and anxiety were compared between the groups. When compared with participants without MSK pain, participants with MSK pain had higher anxiety levels (HADS‐A, 7.00 [4.00–11.00] vs. 4.00 [2.00–6.00], *p* < 0.001). Patients with fluctuation‐related pain had more motor complications than those without fluctuation‐related pain (SCOPA‐Motor Part C 4.50 [2.00–7.75] vs. 1.00 [0.00–2.00], *p* < 0.001). In participants with nocturnal pain, worse sleep complaints, compared with participants without nocturnal pain, were found (NMSS D2: 15.00 [9.50–22.00] vs. 7.00 [2.00–14.00], *p* < 0.001) Table 3.

**TABLE 3:** Differences between the groups formed based on the presence or absence of each of the pain subtypes regarding the severity of motor complications, sleep dysfunction/fatigue and anxiety.

	MSK+ (*n* = 87) vs. MSK− (*n* = 22)	Chronic+ (*n* = 27) vs. Chronic− (*n* = 82)	FRP+ (n = 48) vs. FRP− (*n* = 61)	Nocturnal + (*n* = 41) vs. Nocturnal− (*n* = 68)	D; O/S+ (*n* = 18) vs. D; O/S+ (*n* = 91)	Radicular+ (*n* = 38) vs. Radicular− (*n* = 71)
**SCOPA‐Motor Part C** *Motor complications*	2.00 (0.00–4.00) vs. 2.00 (0.00–5.25) *p* = 0.51	2.00 (0.00–5.00) vs. 2.00 (0.00–4.25) *p* = 0.99	*4.50 (2.00–7.75) vs. 1.00 (0.00–2.00) ***p* < 0.001**	3.00 (1.00–6.50) vs. 2.00 (0.00–3.00) *p* = 0.003	5.00 (1.75–8.00) vs. 2.00 (0.00–4.00) *p* = 0.007	2.00 (0.00–5.25) vs. 2.00 (0.00–4.00) *p* = 0.98
**NMSS D2** *Sleep/fatigue*	12.00 (5.00–20.00) vs. 3.00 (0.00–10.75) *p* = 0.003	10.00 (4.00–21.00) vs. 10.00 (2.00–16.00) *p* = 0.33	12.00 (4.25–20.75) vs. 8.00 (2.50–15.00) *p* = 0.04	*15.00 (9.50–22.00) vs. 7.00 (2.00–14.00) ***p* < 0.001**	16.00 (6.75–26.00) vs. 9.00 (3.00–16.00) *p* = 0.02	9.50 (3.75–20.25) vs. 10.00 (3.00–16.00) *p* = 0.52
**HADS‐A** *Anxiety*	*7.00 (4.00–11.00) vs. 4.00 (2.00–6.00) ***p* < 0.001**	8.00 (4.00–12.00) vs. 6.00 (3.75–9.00) *p* = 0.02	6.50 (4.25–11.00) vs. 6.00 (3.00–8.00) *p* = 0.11	8.00 (5.00–11.00) vs. 6.00 (3.00–8.75) *p* = 0.03	8.00 (6.00–11.25) vs. 6.00 (3.00–9.00) *p* = 0.007	7.50 (4.75–11.00) vs. 6.00 (3.00–8.00) *p* = 0.02

*Note*: Because of the small sample size, KPPS Domain 5 (orofacial pain) is excluded from the analysis. Considering the abnormal distribution noted for most data analysed, all data is displayed as median and interquartile range, and in‐between‐the groups comparisons were run using the non‐parametric Mann–Whitney test. After applying Bonferroni correction, statistically significant differences were set at *p* < 0.0023.

Abbreviations: D; O/S, discoloration, oedema/swelling related pain; FRP, fluctuation‐related pain; HADS‐A, Hospital Anxiety and Depression Scale, sub‐score for anxiety; MSK, Musculoskeletal pain; NMSS, Non‐motor Symptoms Scale; SCOPA‐Motor, SCales for Outcomes in Parkinson's disease—Motor Function.

## 4 DISCUSSION

This explorative cross‐sectional analysis examined potential links between PD‐related pain and other common motor and non‐motor features of PD with an aim to inform personalized, tailored approaches to analgesia in PwP. Our main finding is the significant impact of motor complications, anxiety and sleep disturbances/fatigue on overall burden of PD‐related pain.

Potential mechanisms driving the observed impact of certain PD‐related features on pain in PwP are likely complex and multiple. In our study, motor complications significantly impacted PD‐related pain. Clearly, pain may be directly attributable to motor symptoms associated with levodopa‐induced motor fluctuations (e.g. peak‐dose or biphasic dyskinesia and off‐period dystonia) (Aquino & Fox, 2015). However, although pain in PwP fluctuates with motor oscillations, the severity of these fluctuations does not correlate with the amplitude of motor changes, as demonstrated using a structured interview combined with VAS pain ratings in a study with 100 PwP (Storch et al., 2013). It is possible that PD‐related pain arises consequently to pharmacologically induced chronic phasic stimulation of postsynaptic dopaminergic receptors (Schapira et al., 2017) (Rukavina et al., 2021). Of note, our recent study with 53 PwP (30.2% female; age: 63.79 ± 11.31 years) revealed an association between the presence of PD‐related pain and the severity of dopaminergic deficiency in the caudate nucleus (demonstrated using striatal dopamine transporter visualization with a 123I‐Ioflupane injection and a single‐photon emission computed tomography), further supporting the previously reported involvement of dopaminergic neurotransmission in the modulation of nociceptive information in PwP (Rukavina et al., 2023) (Brefel‐Courbon et al., 2005). Interestingly, however, in a recent randomized controlled trial, adjustments of the daily dose of levodopa/benserazide and oxycodone add‐on both failed to relieve parkinsonian central pain (defined using the algorithm for subtypes identification and classification of chronic pain in PD), implying the likely involvement of other mechanisms in the pathogenesis of this type of PD‐related pain (Marques et al., 2019) (Brefel‐Courbon et al., 2024).

Numerous NMS that are frequently present in PwP may negatively interact with an experience of pain and aggravate its burden, often in a bi‐directional manner. Another significant determinant of PD‐related pain in our study, anxiety, may be implicated in the modulation of the excitability of nociceptive neurons in the dorsal horn, descending and ascending corticospinal tracts and cortical structures, and may have a role in processes leading to hypersensitization (Vidor et al., 2014). In addition, sleep disturbances are highly comorbid with chronic pain (Finan et al., 2013; Stefani & Hogl, 2020). Large population‐based studies (in non‐PD affected individuals) identified poor sleep quality and insufficient sleep duration as risk factors for the development of chronic pain. Strong evidence that short or disturbed sleep may cause hyperalgesia (an increased sensitivity to painful stimulation) and give rise to the development or worsening of spontaneous pain symptoms is available from numerous studies using experimental models of sleep loss, but a detailed discussion on respective studies is beyond the scope of this manuscript (Haack et al., 2020) (Whibley et al., 2019).

It is possible that, in PwP, pain, anxiety and sleep disturbances share common pathophysiological substrates. For example, these symptoms may arise from neuroplastic maladaptation involving the locus coeruleus (LC, the major producer of noradrenaline in the central nervous system); such functional changes may occur early in the course of PD (Schapira et al., 2017) (Morris et al., 2020) (Paredes‐Rodriguez et al., 2020). In health, noradrenergic transmission from the LC exerts a pain‐modulatory effect (Kucharczyk et al., 2022) (Taylor & Westlund, 2017). The role of the LC in chronic pain generation relates to its distinct modular organization, whereby both descending and ascending projections have been reported to act counter‐intuitively to the traditionally viewed ‘pain inhibiting’ role of the LC (Taylor & Westlund, 2017) (Kucharczyk et al., 2022). LC dysregulation (reduced auto‐inhibition and increased tonic activity after stimulation) has been linked to excessive responses to threat, maladaptive exaggerated fear and pathological anxiety responses (Morris et al., 2020) (Paredes‐Rodriguez et al., 2020), as well as pain.

PD‐related pain has been proposed as a key feature dominating the recently described noradrenergic subtype of PD (Ray Chaudhuri et al., 2023). Clinically, this is underpinned by observations made in previous studies: A post hoc analysis of the King's Parkinson's Disease Pain Questionnaire Validation Study (a cross‐sectional study enrolling 300 PwP, 40.3% women, mean age 64.9 ± 10.5 years) used a multiple regression model, where, following an adjustment for other confounders, MSK and nocturnal PD‐related pain (defined based on the KPPS) were significantly associated with nocturnal sleep disorders (measured using the Parkinson's Disease Pain Scale −2, PDSS‐2) (Martinez‐Martin et al., 2019). In addition, in a recently published dual‐centre cross‐sectional study (*n* = 167 PwP, 37.1% women, mean age 66.1 ± 9.5 years), an independent association between the presence of PD‐related pain (captured as KPPS total score ≥1), sleep impairment and cardiovascular abnormalities was noted (Ghosh et al., 2020). Similarly, a multi‐partition clustering model (402 PwP, median Hoehn and Yahr stage 2 [1–4]) revealed an association between PD‐related pain (captured using the Movement Disorder Society Non‐Motor Rating Scale, MDS‐NMS) and sleep, gastrointestinal and urinary symptoms, apathy and cognitive disturbances (Rodriguez‐Sanchez et al., 2021). Of note, in contrast to previous reports, here we did not observe the co‐occurrence of PD‐related pain and cardiovascular symptoms. This may be explained by a low burden of cardiovascular symptoms in our participants, possibly causing a floor effect (Appendix S1).

Our study has certain limitations, most importantly the discrepancy between the time period of pain presence assessed by the KPPS (1 month) and the International Classification of Diseases, Eleventh Revision (ICD‐11) definition of chronic pain (pain that persists or recurs for longer than 3 months), alongside the lack of information on exact pain duration in this dataset (ICD‐11) (Mylius et al., 2024). Despite this lack of congruence, the use of KPPS for intensity rating and syndromic classification of PD‐related pain is recommended by the International Parkinson and Movement Disorders Society (MDS) Steering Committee on Rating Scale Development and is in keeping with numerous clinical trials globally (Perez‐Lloret et al., 2016) (Kurihara et al., 2022) (Coimbra et al., 2021) (Behari et al., 2020) (Gao et al., 2022) (Jost et al., 2018) (Stoyanova‐Piroth et al., 2021) (Trenkwalder et al., 2015) (Rascol et al., 2016) (Grigoriou et al., 2021) (Geroin et al., 2020) (Santos Garcia et al., 2021) (Yu et al., 2019) (Alissa et al., 2023) (Joseph et al., 2023). Secondly, due to the NILS' key aim, gathering of ‘real‐life’ data on NMS burden in individuals with PD, participants were approached while attending their routine appointments if inclusion criteria were met and no exclusion criteria were present (as estimated by their attending neurologists), rather than being invited through a formal screening process. While, consequently, an exact number of screened participants and of those excluded remains unknown, the important value of this approach is that the collected data reflects clinical reality more accurately (van Wamelen et al., 2021) (Dafsari et al., 2019). Another important limitation is the fact that the most common type of PD‐related pain, musculoskeletal pain (affecting 80% of participants in our study), is highly prevalent in older adults, with an overall prevalence of around 35% (Cimas et al., 2018) (Ghosh et al., 2020). Of note, the two are different: PD‐related musculoskeletal pain, in contrast to musculoskeletal pain in general population, is dopamine‐responsive in most affected PwP (e.g. in a recent study, MSP was responsive to Levodopa resulting in at least 30% reduction of pain severity on numeric rating scale in 82.52% participating PwP), allowing for clinical differentiation (Li et al., 2021). Nevertheless, our study would benefit from a control group of individuals unaffected by PD to compare the burden of musculoskeletal pain. Alternatively, a recently published framework to differentiate PD‐ from non‐PD‐related pain by Mylius et al. could have been used in a structured way (Mylius et al., 2021).

To conclude, our findings outline the interrelations of PD‐related pain with motor complications, anxiety and sleep disturbances. Despite the limitations of this explorative, retrospective analysis, our findings have a potential to accelerate the delivery of precision pain management for PwP, allowing for a personalized, tailored approach which will rely on accurate recognition and management of motor and NMS interconnected with PD‐related pain. Whether successful management of motor complications, anxiety and sleep disturbances may contribute to pain relief in PwP for specified cohorts warrants future randomized controlled clinical trials.

## AUTHOR CONTRIBUTIONS

Design: KR, KRC and KB. Execution and data collection: KR, JS, PZ, KW and AR. Analysis: KR and MKS. Review: KB, KPD and KRC. Writing of the first draft: KR. Editing of final version of the manuscript: All authors.

## Supporting information


Appendix S1.
